# Widespread overexpression from the four DNA hypermethylated HOX clusters in aggressive (*IDH*wt) glioma is associated with H3K27me3 depletion and alternative promoter usage

**DOI:** 10.1002/1878-0261.12944

**Published:** 2021-05-02

**Authors:** Elisa Le Boiteux, Franck Court, Pierre‐Olivier Guichet, Catherine Vaurs‐Barrière, Isabelle Vaillant, Emmanuel Chautard, Pierre Verrelle, Bruno M. Costa, Lucie Karayan‐Tapon, Anne Fogli, Philippe Arnaud

**Affiliations:** ^1^ CNRS Inserm GReD Université Clermont Auvergne Clermont‐Ferrand France; ^2^ INSERM‐U1084 Poitiers France; ^3^ Poitiers University France; ^4^ Department of Cancer Biology Poitiers Hospital France; ^5^ Pathology Department Jean Perrin Center Clermont‐Ferrand France; ^6^ INSERM U1240 IMoST Université Clermont Auvergne Clermont‐Ferrand France; ^7^ CIMB INSERM U1196 CNRS UMR9187 Curie Institute Orsay France; ^8^ Radiotherapy Department Curie Institute Paris France; ^9^ Université Clermont Auvergne Clermont‐Ferrand France; ^10^ Life and Health Sciences Research Institute (ICVS) School of Medicine University of Minho, Campus de Gualtar Braga Portugal; ^11^ ICVS/3B's – PT Government Associate Laboratory Braga/Guimarães Portugal; ^12^ Biochemistry and Molecular Biology Department Clermont‐Ferrand Hospital France

**Keywords:** alternative promoters, cancer, epigenetics, glioma, glioma stem cells, HOX genes

## Abstract

In human, the 39 coding HOX genes and 18 referenced noncoding antisense transcripts are arranged in four genomic clusters named HOXA, B, C, and D. This highly conserved family belongs to the homeobox class of genes that encode transcription factors required for normal development. Therefore, HOX gene deregulation might contribute to the development of many cancer types. Here, we study HOX gene deregulation in adult glioma, a common type of primary brain tumor. We performed extensive molecular analysis of tumor samples, classified according to their isocitrate dehydrogenase (*IDH1*) gene mutation status, and of glioma stem cells. We found widespread expression of sense and antisense HOX transcripts only in aggressive (*IDH*wt) glioma samples, although the four HOX clusters displayed DNA hypermethylation. Integrative analysis of expression, DNA methylation, and histone modification signatures along the clusters revealed that HOX gene upregulation relies on canonical and alternative bivalent CpG island promoters that escape hypermethylation. H3K27me3 loss at these promoters emerges as the main cause of widespread HOX gene upregulation in *IDH*wt glioma cell lines and tumors. Our study provides the first comprehensive description of the epigenetic changes at HOX clusters and their contribution to the transcriptional changes observed in adult glioma. It also identified putative ‘master’ HOX proteins that might contribute to the tumorigenic potential of glioma stem cells.

## Graphical abstract

This study describes the epigenetic and transcriptional changes at HOX clusters in aggressive glioma. It shows how DNA hypermethylation and gene overexpression can coexist and suggest that loss of H3K27me3 along the HOX clusters is the main driving force of HOX widespread transcriptional alteration in aggressive glioma samples. It highlights the complexity of HOX gene expression pattern in patients where the usage of alternative promoters contributes to splice variant variability among samples.

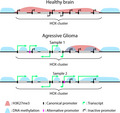

**Abbreviations**
CGI: CpG islands
CNV: copy number variation
EZH2: enhancer of zeste homolog 2
FPKM: fragment per kilobase per million reads
GBM: glioblastoma
GSC: glioma stem cells
H3K27ac: acetylation of histone H3 lysine 27
H3K27me3: trimethylation of histone H3 lysine 27
H3K36me3: trimethylation histone H3 lysine 36
H3K4me3: trimethylation of histone H3 lysine 4
IDH: isocitrate dehydrogenase
*IDH*mut: mutated *IDH1* genes
*IDH*wt: wild‐type *IDH1* and *IDH2* genes
TSS: transcription start site

## 1 Introduction

Glioma is the most frequent primary malignant brain tumor worldwide, with more than 200 000 cases per year. Although glioma is mainly diagnosed in 45‐ to 65‐year‐old individuals, it is the second cause of death by cancer in children and the third in young adults. Until recently, diffuse glioma was classified into three grades, grade II to highly malignant grade IV or glioblastoma (GBM), mainly based on their microscopic features [1]. The last released 2016 World Health Organization (WHO) classification [2], used in this study, takes also into account molecular features, which are primarily based on the isocitrate dehydrogenase (*IDH*) gene mutation status. Specifically, *IDH1* and *IDH2* are wild‐type (*IDH*wt) in the most aggressive glioma types, which include about 95% of GBM, whereas they are mutated (*IDH*mut) in gliomas with better prognosis [3]. Although aggressive treatments (surgery, chemotherapy, and radiotherapy) are commonly used, *IDH*wt gliomas are recurrent and almost always fatal. The median survival time after diagnosis of *IDH*wt GBM does not exceed 18 months. It is thought that therapeutic resistance and tumor relapse are mainly caused by a cell subpopulation with stem cell characteristics, called glioma stem cells (GSCs) [4, 5]. Therefore, it is important to determine the molecular bases of GSC oncogenic potential to improve *IDH*wt glioma management.

The highly conserved HOX family belongs to the homeobox class of genes that encode transcription factors required for normal development. In mammals, HOX genes are arranged in four paralogous genomic clusters, named A, B, C, and D. In the human genome, these clusters are located on chromosome 7, 17, 12, and 2, respectively, and contain 39 coding HOX genes and 18 referenced noncoding antisense transcripts. During embryogenesis, their activation in a temporal and spatial collinear manner, depending on their location within the cluster, is critical for body patterning [6]. Besides embryo development, HOX genes exert other functions, for instance in late fetal/early postnatal brain development [7]. The temporally and spatially regulated induction of HOX genes relies on a multiscale mechanism that involves *cis*‐regulatory elements, the three‐dimensional chromatin conformation, chromatin boundaries, and histone modifications [8]. Specifically, this process is resumed by the deposition of the repressive H3K27me3 and the active H3K4me3 marks along the cluster by the polycomb (PcG) and trithorax (TrxG) groups of proteins, respectively.

HOX gene deregulation contributes to cancer development. Aberrant HOX gene/protein expression is a hallmark of leukemia and of many solid cancer types, such as breast [9], bladder ([10], kidney [11], and brain tumors, where HOX proteins can have oncogenic or tumor suppressor roles [12, 13]. In glioma and particularly in GBM, many HOX factors are upregulated (transcript and protein level), suggesting that all four clusters are deregulated [14, 15, 16, 17, 18]. However, these findings are based on studies performed using different tumor samples or cancer cell lines and different methods to assess the expression. Therefore, the precise extent of this deregulation in glioma remains to be determined [18]. Functional analyses suggest that at least 18 of the 39 HOX coding genes have a functional role in glioma, acting mostly as oncogenes [18]. Aberrant activation of HOX genes has been observed also in GSCs where it has been functionally associated with their oncogenic potential [19].

Several molecular alterations might contribute to aberrant HOX gene expression in glioma, including epigenetic alterations and gene copy number variations (CNV). Specifically, *IDH*wt samples are characterized by chromosome 7 gain and chromosome 10 loss [2], whereas *IDH*mut samples mainly harbor the 1p/19q codeletion. As the HOXA locus is on chromosome 7, gains at this locus might increase HOXA gene expression in these patients (reviewed in Ref. [18]). Concerning epigenetic alterations, aberrant gain of DNA methylation is a feature of HOX loci in GBM [20, 21]. However, given its inhibitory effect on gene promoter activity, it is not clear whether and how DNA methylation contributes to HOX gene deregulation [22]. Alterations in the pathways that control H3K27me3 and H3K4me3 deposition also could be involved in HOX gene deregulation. Specifically, in GSCs, mixed lineage leukemia (MLL), a trithorax protein, is required for *HOXA10* activation that in turn activates a network of downstream genes, including other HOX genes, contributing to GSC tumorigenic potential [19]. Moreover, in GBM cell lines, the PI3K pathway, which mediates phosphorylation of EZH2, the catalytic subunit of the polycomb PRC2 complex, contributes to HOXA cluster derepression, through suppression of H3K27me3 as shown for *HOXA9* [16]. Similarly, we recently showed that HOX genes belong to a discrete class of genes characterized by ectopic expression, DNA hypermethylation, and H3K27me3 loss in *IDH*wt tumors [22].

Altogether, these observations suggest that the mechanism underlying HOX gene deregulation in glioma is complex and that the main driving force remains to be determined. Therefore, we carried out an extensive molecular analysis of *IDH*mut and *IDH*wt glioma samples and integrative molecular analyses in GSC lines.

## 2 Methods

### 2.1 Tumor and control samples

Adult diffuse glioma samples (*n* = 70), resected between 2007 and 2014, were obtained from Clermont‐Ferrand University Hospital Center, France (‘Tumorotheque Auvergne Gliomes’, ethical approval DC‐2012‐1584). This study was approved by the relevant ethics committees and competent authorities, and the study protocols follow the World Medical Association Declaration of Helsinki. Samples were isolated as previously described [22]. Briefly, they were snap‐frozen immediately after surgery and stored in liquid nitrogen. Necrosis extent and tumor cell percentage were determined by analysis of random sections from each tumor sample under a light microscope after hematoxylin/eosin staining. All glioma samples used for this study contained at least 50% of tumor cells. Tumors were classified according to their *IDH1* mutation status: *IDH*wt (*n* = 55) and *IDH*mut (*n* = 15), following the 2016 WHO classification [2]. Patients with *IDH*mut tumors showed better survival [HR = 0.32, 95% CI (0.14–0.71), *P* = 0.005] [22]. Among the *IDH*wt samples, only two harbored a 1p/19q codeletion (a good prognostic marker) (Table S1). The patients' demographic and clinical features are presented in Table S1.

Fifteen control brain samples (healthy controls; samples removed by autopsy 4–16 h after accidental death) were obtained from the Brain and Tissue Bank of Maryland (mean age of 27.3 years, standard deviation ± 2 years). These samples, identified by the Brain and Tissue Bank of Maryland as corpus callosum (*n* = 8) and frontal cortex (*n* = 7), correspond to white matter enriched in astrocytes and oligodendrocytes and are relevant noncancer controls for gliomas. Before use, each tumor and control sample was homogenized into powder by cryogenic grinding and distributed in at least three vials for genomic DNA, RNA, and chromatin extraction. All samples were stored at −80 °C until use.

Cell pellets from eight GSC lines (GSC‐1, GSC‐2, GSC‐3, CSG‐5, GSC‐6, GSC‐9, CSG‐10, and GSC‐11) derived from patients with *IDH*wt GBM were obtained from Poitiers University Hospital Centre, France and were previously characterized [23, 24, 25].

Validation cohorts, obtained from The Cancer Genome Atlas (TCGA) research network, were described in [26]. For this study, *IDH*mut (*n* = 415) and *IDH*wt (*n* = 134) samples with both DNA methylation (HM450K array) and RNA expression (RNA‐seq) data were selected. The clinical and molecular data of these patients were retrieved from cBioPortal for Cancer Genomics (https://www.cbioportal.org/) [27, 28]. Processed RNA‐seq and methylation data were obtained from the TCGA website (https://portal.gdc.cancer.gov/) and analyzed as described below.

### 2.2 Expression analysis

#### 2.2.1 RNA extraction

Total RNA was isolated from frozen tissue samples and frozen cell pellets using the RNeasy Mini Kit (Qiagen, Courtaboeuf, France) and treated with DNase I (Promega, Charbonnières‐les‐Bains, France), according to the manufacturer's recommendations. RNA quality was evaluated using a Bioanalyzer (Agilent) or a TapeStation system (Agilent, Les Ulis, France). Only samples with RIN > 6 were retained for expression analysis (*IDH*wt glioma *n* = 43, *IDH*mut glioma *n* = 8, controls *n* = 10, GSC *n* = 6). All RNA samples were stored at −80 °C until use.

#### 2.2.2 Strand‐oriented RNA‐seq

Strand‐oriented RNA‐seq was performed with total RNA [*n* = 3 brain control, *n* = 8 *IDH*wt, *n* = 5 *IDH*mut, and *n* = 2 GSC samples (CSG‐1 and CSG‐2)] and also with mRNA from the GSC‐6 and GSC‐11 samples. Sequencing data were analyzed as previously described [22]. Briefly, RNA‐seq data were mapped to the hg19 human genome assembly using tophat2 (version 2.1.0) (https://ccb.jhu.edu/software/tophat/index.shtml) and a transcript annotation file from GENCODE (Release 19). The read count per gene was obtained with the HTseq‐count script. Strand‐specific RNA‐seq coverage was obtained using the samtools (v 1.9) ( https://github.com/samtools/samtools), genomecoveragebed (v2.27.1) (https://bedtools.readthedocs.io/en/latest/content/tools/genomecov.html), and bedgraphtobigwig (http://hgdownload.soe.ucsc.edu/admin/exe/linux.x86_64/bedGraphToBigWig) tools and visualized using the UCSC Genome Browser. Differential expression analyses were based on read counts using the deseq2 (https://bioconductor.org/packages/release/bioc/html/DESeq2.html) and edger r(3.6) (https://bioconductor.org/packages/release/bioc/html/edgeR.html) packages. Genes were considered as differentially expressed between groups when |log_2_(fold change)| > 1 with an adjusted *P*‐value < 0.05 in both statistical approaches. Genes located on the chromosomes X, Y, and M were excluded from the analysis. Raw data are accessible at GSE123892, GSE161438, and GSE161437.

#### 2.2.3 RT‐qPCR analyses

Two independent reverse transcription (RT) reactions per sample (250 ng RNA/reaction) were performed using SuperScript IV (Invitrogen‐Fisher Scientific, Illkirch, France) and random primers (Invitrogen), according to the manufacturer's recommendations. Real‐time quantitative PCR (qPCR) assays were performed using a microfluidic‐based approach by the Gentiane facility (INRA Crouël, Clermont‐Ferrand, France). Briefly, 5 µL of cDNA (diluted to 1/2.5) was pre‐amplified (14 cycles) with the primer pool and the TaqMan PreAmplification Master Mix (Invitrogen‐Fisher Scientific, Illkirch, France). After digestion using an exonuclease (NEB, Evry, France) to remove the remaining primers, qPCR was performed on Fluidigm 96.96 Dynamic Arrays using the Biomark HD system (Fluidigm Corp., Les Ulis, France) according to the manufacturer's instructions. The relative expression level was calculated as follows: *E* ^−Ct(Transcript)^/geometrical mean (*E* ^−Ct(HK genes)^), based on the 2^−ΔCt^ method (*E*: efficiency of amplification, Ct: cycle threshold, HK: housekeeping). The housekeeping genes *PPIA*, *TBP*, and *HPRT1* were used to normalize transcript expression. For each condition, the presented data were obtained from the two independent RT reactions, each analyzed in duplicate using the fluidigm real‐time pcr analysis software (Fluidigm Corp., San‐Francisco, CA, USA). Samples with an unexpected Tm or with a double melting peak were excluded from the analysis. All samples with a Ct > 21 for a gene were considered as lacking expression of that gene. Genes were considered as differentially expressed between groups when the *P*‐value was < 0.05 (determined with the Wilcoxon signed‐rank test). The primers used are described in Table S2.

### 2.3 Copy number variation analyses

CNV analyses were performed using 3 control, 36 *IDH*wt glioma and 12 *IDH*mut glioma samples, and Genome‐Wide Human CytoScan HD (Affymetrix), as previously described [22]. Raw data are accessible at GSE123682 and GSE161275. HOX gene expression was determined using the microfluidic approach described above. Correlations between CNV and expression data (available for 21 *IDH*wt samples) were analyzed with the Spearman rank correlation test.

### 2.4 DNA methylation analysis

#### 2.4.1 DNA extraction

DNA was isolated from frozen tissue samples and frozen cell pellets using the QIAamp DNA Mini Kit (Qiagen) according to the manufacturer's recommendations. DNA purity and concentration were determined with a Nanodrop spectrophotometer (Thermo Scientific, Illkirch, France). All DNA samples were stored at −20 °C until use.

#### 2.4.2 Array‐based DNA methylation analysis

DNA from 78 samples (*n* = 55 *IDH*wt glioma, *n* = 15 *IDH*mut glioma, *n* = 8 control samples) was analyzed using Infinium Human Methylation 450k (Illumina), and DNA from GSC samples (*n* = 2) was analyzed using Infinium Human Methylation EPIC (Illumina). DNA bisulfite conversion and array hybridization were performed by IntegraGen, SA (Evry, France) using the Illumina Infinium HD methylation protocol (Illumina, San Diego, CA, USA). Analyses were performed as previously described [29]. Raw data are accessible at GSE123678 (HM450K data) and GSE161175 (EPIC data).

DNA methylation and expression data were correlated using data from 43 *IDH*wt samples of our cohort and from 134 *IDH*wt samples of the TCGA cohort. The DNA methylation level of CpG sites was correlated with the gene expression level in the HOX clusters using Spearman's rank correlation coefficient.

### 2.5 Chromatin analyses

#### 2.5.1 Chromatin immunoprecipitation‐qPCR of glioma samples

Antibodies against histone H3 acetylated at lysine 9 (H3K9ac) (Millipore 06‐942) (Millipore, St‐Quentin‐en‐Yvelines, France), H3K4me3 (Diagenode 03‐050) (Diagenode, Liège, Belgium), and H3K27me3 (Millipore 07‐449) were used to assess the enrichment of these histone marks at selected genes by chromatin immunoprecipitation (ChIP) of native chromatin isolated from frozen tissue samples (*n* = 7 *IDH*wt glioma, *n* = 5 *IDH*mut glioma, *n* = 5 control samples), as previously described [30]. These samples were randomly selected among those retained for expression analysis and with sufficient starting material for ChIP analysis. Input and antibody‐bound fractions were quantified by qPCR with the SYBR Green mixture (Roche, Meylan, France) and a LightCycler 480II (Roche) instrument. Background precipitation levels were determined by performing mock precipitations with a nonspecific IgG antiserum (Sigma‐Aldrich C‐2288) (Sigma‐Aldrich/Merck, St. Quentin Fallavier, France) and were only a fraction of the precipitation levels obtained with the specific antibodies. The bound/input ratios were calculated and normalized to the precipitation level at the *TBP* promoter for the ChIP experiments with the anti‐H3K9ac and ‐H3K4me3 antibodies and at the *SP6* promoter for the ChIP experiments with the anti‐H3K27me3 antibody. The primers used are described in Table S2.

#### 2.5.2 ChIP‐seq of GSC samples

ChIP‐seq was performed using native chromatin isolated from frozen GSC pellets (*n* = 2), as described above. After 1‐h incubation in immunoprecipitation buffer, samples underwent an additional centrifugation step (13 000 ***g*** for 10 min) to improve the signal‐to‐noise ratio of the sequencing data. For ChIP, antibodies against histone H3 acetylated at lysine 27 (H3K27ac) (Abcam Ab4729) (Abcam, Paris, France), H3K4me3 (Diagenode 03‐050), and H3K27me3 (Millipore 07‐449) were used. Background precipitation levels were determined by performing mock precipitations with a nonspecific IgG antiserum (Sigma‐Aldrich C2288), and experiments were validated by qPCR before sequencing. Library preparation and sequencing on a NovaSeq 6000 instrument (Illumina) were performed by IntegraGen SA, according to the manufacturer's recommendations (mean of 20 million paired reads per sample). ChIP‐seq reads of replicate 1 (R1) were mapped to the human genome (hg19) using the bowtie2 (v 2.3.4.3) (http://bowtie‐bio.sourceforge.net/bowtie2/index.shtml) tool. Alignments were filtered according to their quality (Mapq > 30) using SamTools (v 1.9). ChIP‐seq signals were generated with Bamcoverage (v 3.1.3) (options: normalizeUsing RPKM, extendReads 200, ignoreDuplicates, binSize 20) and visualized with UCSC Genome Browser. Peaks were detected with macs (v 1.4.2) (https://github.com/taoliu/MACS/archive/v1.4.2.tar.gz), using Input as control (options: nomodel shiftsize 73, for H3K27ac and H3K4me3 *P* value = 1e^−5^, for H3K27me3 *P* value = 1e^−3^).

*K*‐means clustering of H3K4me3 TSS enrichment was performed with the computeMatrix and plotHeatmap tools from the deeptools (3.1.3) (https://deeptools.readthedocs.io/en/develop/index.html) suite and the GSM1121865 coverage data. Raw data are accessible at GSE161436.

#### 2.5.3 ChIP‐seq data mining associated with chromatin analyses

ChIP‐seq data for the H3K4me3, H3K27me3, and histone H3 methylated at lysine 36 (H3K36me3) profiles were obtained from the NIH Roadmap Epigenomics project (http://www.roadmapepigenomics.org/) (neural progenitor cells, NPCs, and brain samples) or from the GEO database (glioblastoma and GSC samples), as follows: NPC samples (H3K4me3: GSM818043, GSM772736. H3K27me3: GSM956010, GSM772801. H3K36me3: GSM772795, GSM1013141), brain samples (H3K4me3: GSM772996, GSM669992. H3K27me3: GSM772772, GSM772993. H3K36me3: GSM670011, GSM772982), GBM samples (H3K4me3: GSM1121865, GSM1121875, GSM1121888. H3K27me3: GSM1121862, GSM1121872, GSM1121885. H3K36me3: GSM1121863, GSM1121873, GSM1121886), and GSC samples (H3K4me3: GSM1121860, GSM1121870, GSM1121882. H3K27me3: GSM1121857, GSM1121867, GSM1121879. H3K36me3: GSM1121858, GSM1121868, GSM1121880).

### 2.6 Functional annotations

Regulatory features and *cis*‐regulatory modules were predicted using i‐*cis* Target [31]. This tool allows analyzing the regulatory regions of gene lists to detect enrichment of transcription factor binding sites [TFBSs; i.e., consensus DNA sequences to which a transcription factor binds and represented as position weight matrices (PWM)]. Only motifs with a normalized enrichment score (NES) above a specified threshold (here 3.0) are considered enriched. More details are provided at the i‐*cis* Target website: (https://gbiomed.kuleuven.be/apps/lcb/i‐cisTarget/) (v5.0). PWM data (*n* = 20 003) for the promoter (defined as ±1 kbp of RefSeq TSS) of overexpressed and not affected HOX genes were analyzed.

## 3 Results

### 3.1 Widespread reactivation of HOX genes in *IDH*wt tumors

For this study, we used 70 primary adult diffuse glioma samples classified according to their *IDH*1 mutation status (*n* = 55 *IDH*wt and *n* = 15 *IDH*mut) (described in Ref. [22], and Table S1), and six *IDH*wt GSC lines.

**Fig. 1:**
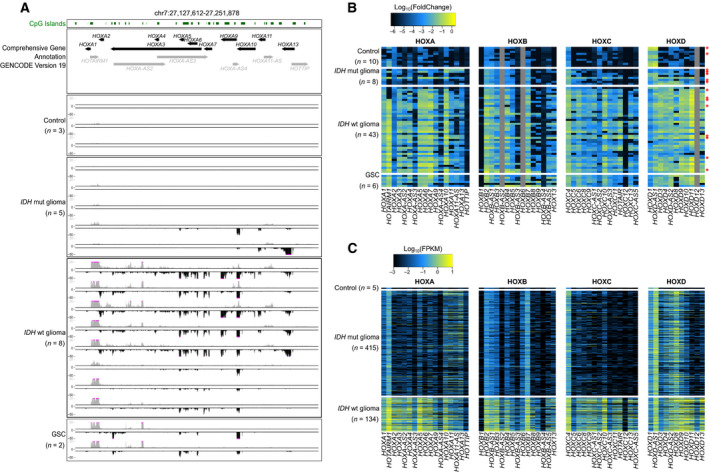
Widespread reactivation of HOX transcripts in *IDH*wt glioma samples. (A) Strand‐oriented RNA‐seq signals along the HOXA cluster in control (*n* = 3), *IDH*mut (*n* = 5) and *IDH*wt (*n* = 8) glioma and Glioma stem cells (GSC) (*n* = 2) samples. For each sample, sense (in black) and antisense (in gray) transcription signals are shown in the lower and upper panels, respectively. (B, C) Relative expression level of HOX coding and noncoding transcripts in control, *IDH*mut and *IDH*wt glioma, and GSC samples from our cohort analyzed by microfluidic‐based RT‐qPCR (B) and from the TCGA cohort, analyzed by RNA‐seq (C). In (B), values are the fold change relative to the geometrical mean of expression of the housekeeping genes *PPIA*, *TBP*, and *HPRT1*. In our cohort, expression of *HOXB‐AS2*, *HOXB6,* and *HOXD12* was not analyzed. Samples analyzed both by RNA‐seq (A) and microfluidic‐based RT‐qPCR (B) are indicated with a red star in (B). FPKM, fragment per kilobase per million reads.

We first assessed HOX gene expression in three normal brain tissue (control) samples, eight *IDH*wt and five *IDH*mut glioma samples, and two GSC lines using a strand‐oriented RNA‐seq approach to analyze independently sense and antisense transcripts. Most of the genes in the HOXA cluster were not expressed in control and *IDH*mut samples. Conversely, we observed widespread but heterogeneous reactivation of coding and noncoding antisense transcripts in the *IDH*wt samples and GSC lines (Fig. 1A). We obtained similar results also for the other three HOX clusters (Fig. S1), indicating that overall, HOX genes are expressed in *IDH*wt glioma samples and *IDH*wt GSC lines.

RT‐qPCR analysis (*n* = 10 control samples, *n* = 8 *IDH*mut and *n* = 43 *IDH*wt glioma samples, and *n* = 6 GSC lines) confirmed the widespread reactivation of HOX genes in *IDH*wt glioma samples and GSC lines (Fig. 1B). More than 80% of HOX transcripts were upregulated in most *IDH*wt glioma samples compared with control samples: 32 of the 37 coding and 12 of the 17 noncoding transcripts analyzed (Table S3). A dozen of them, mainly from the HOXA and HOXD clusters, were upregulated in more than 85% of *IDH*wt glioma samples. Conversely, other transcripts, such as *HOXA‐AS3*, *HOXB‐AS3* and *HOXB9,* were upregulated only in approximately 20% of *IDH*wt glioma samples (Fig. 1B and Table S4). Finally, two coding (*HOXB1*, *HOXC12*) and two noncoding transcripts (*HOTTIP* and *HOXB4‐AS4*) remained repressed in all analyzed samples (Fig. 1, Table S4).

To assess the reproducibility of these observations, we performed the same analyses in an independent cohort (‘TCGA cohort’) that included 5 control, 415 *IDH*mut, and 134 *IDH*wt glioma samples [26]. We could confirm the widespread reactivation of HOX coding (36/39) and noncoding transcripts (13/18) in *IDH*wt glioma compared with control samples. Moreover, several of them (e.g., *HOXA1, A5, A6, A7, A10, D9,* and *D10*) were expressed in most *IDH*wt glioma samples, constituting thus the core HOX signature in *IDH*wt samples, whereas *HOXB1, HOXC12, HOTTIP,* and *HOXB‐AS4* were not reactivated, as shown before (Fig. 1C, Tables S3 and S4).

These data indicate that aberrant HOX gene expression is a feature of *IDH*wt glioma, suggesting a coordinated deregulation process at the four HOX clusters.

### 3.2 Genome rearrangements are not the main cause of HOX gene upregulation

**Fig. 2:**
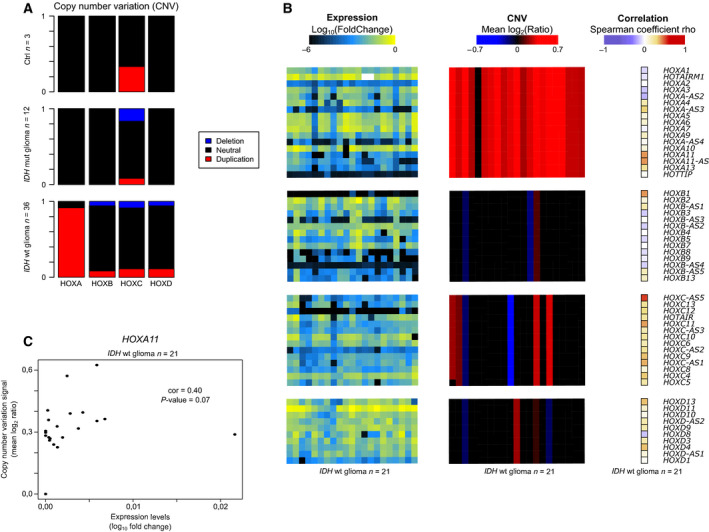
Absence of significant correlation between CNVs and expression of HOX genes. (A) Proportion of duplication (red) and deletion (blue) at the HOXA, B, C, and D clusters in control brain (*n* = 3) and *IDH*wt (*n* = 12) and *IDH*mut (*n* = 36) glioma samples. (B) Correlation analysis between CNV and expression levels at the HOXA, B, C, and D clusters in 21 *IDH*wt glioma samples. (C) CNV and expression correlation analysis for the *HOXA11* gene in 21 *IDH*wt glioma samples (*r* = 0.4; *P*‐value = 0.07).

To determine whether genomic rearrangements, which are often observed in cancer cells, could contribute to HOX gene overexpression, we analyzed CNV in the four HOX clusters. Chromosome 7 gain characterized *IDH*wt samples [2]. Accordingly, about 90% of the *IDH*wt glioma samples analyzed carried extra copies of the HOXA cluster that is located on chromosome 7. Conversely, we did not detect any major rearrangement at the HOXB, C, and D clusters, located on chromosomes 17, 12, and 2, respectively (Fig. 2A). This indicated that gene upregulation in these clusters relies on other mechanisms. Moreover, by comparing CNV and expression data (RT‐qPCR) in 21 samples, we did not detect any significant correlation between CNV and HOXA cluster gene expression. The highest correlation value for the *HOXA11* gene (*r* = 0.4) was not significant (Fig. 2B,C and Table S5), suggesting that chromosome 7 gain contributes but is not the main driving force of HOXA gene upregulation in *IDH*wt glioma samples.

### 3.3 Self‐ and cross‐regulatory interactions between HOX transcription factors and HOX genes could contribute to HOX widespread reactivation in *IDH*wt glioma

**Fig. 3:**
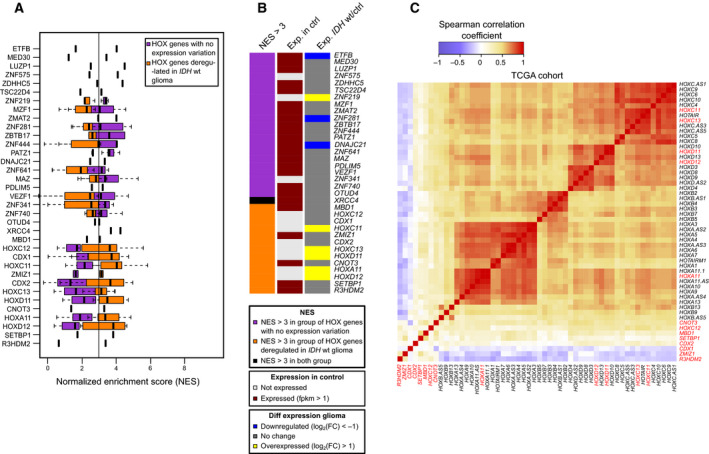
Expression of HOX transcription factors and HOX genes in *IDH*wt glioma samples. (A) Transcription factor motif enrichment in the promoter of HOX genes (defined as ±1 kbp of RefSeq TSS), calculated using i‐*cis* Target and represented as normalized enrichment score (NES). Orange squares, genes upregulated in *IDH*wt glioma; purple squares, genes that are not deregulated in *IDH*wt glioma. When a transcription factor harbors several binding motifs, data are presented as a box plot. (B) Expression status, assessed by RNA‐seq, of the transcription factors identified in A. The middle column shows their expression status in healthy brain controls (*n* = 3) (gray, not expressed; burgundy, expressed: FPKM > 1) and the right column their expression in *IDH*wt glioma samples (*n* = 8) compared with controls (blue, downregulated: log2(FoldChange) < −1; gray, no change; yellow, overexpressed: log2(FoldChange) > 1). The left column shows the motif enrichment specific to HOX genes that are not deregulated (purple) or deregulated (orange) in *IDH*wt glioma and in both categories (black). (C) Heatmap of the correlation between all HOX genes and the transcription factors identified in A (in red), established using publicly available RNA‐seq data from 134 *IDH*wt samples (TCGA cohort). FPKM, fragment per kilobase per million reads.

To test whether the widespread HOX gene overexpression could be due to misregulation of shared transcription factors, we analyzed motif enrichment at promoter regions of HOX genes that were expressed or not in *IDH*wt glioma samples. We found that the binding sites of 13 transcription factors, including six HOX transcription factors, were enriched at 44 HOX transcripts expressed in *IDH*wt glioma samples (Fig. 3A). Most of these transcription factors were similarly expressed between control and *IDH*wt glioma samples. However, five of the six HOX transcription factors were expressed only in glioma samples (Fig. 3B). To determine whether these factors contributed to the HOX gene reactivation, we analyzed the correlation between their expression and that of deregulated HOX genes in *IDH*wt glioma samples using the RNA‐seq data of the 134 *IDH*wt glioma samples from the ‘TCGA cohort’. The expression of the five HOX transcription factors correlated only with that of the other deregulated HOX genes from the same HOX cluster (HOXA, HOXC, and HOXD) (Fig. 3C). This suggests that the initial upregulation of few key HOX genes could lead to reactivation of most genes belonging to the same HOX cluster.

### 3.4 Some HOX gene transcription start sites escape hypermethylation

**Fig. 4:**
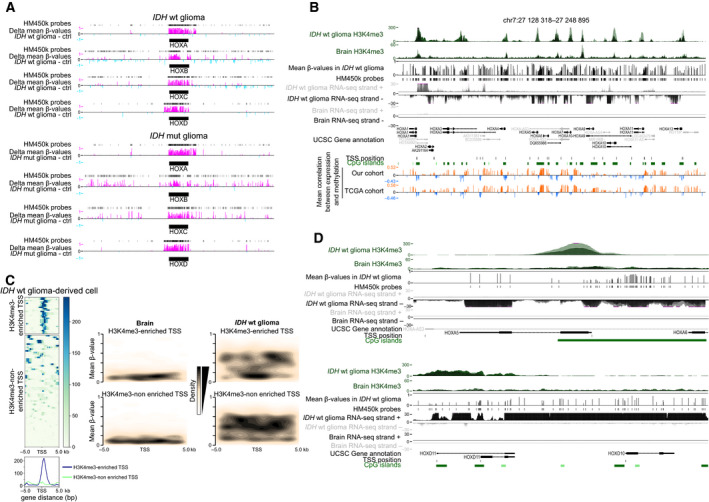
A subset of HOX gene TSS escapes DNA hypermethylation in *IDH*wt glioma samples. (A) DNA methylation changes (compared with control samples) along each HOX cluster and their surrounding genomic regions (±500 kbp) in *IDH*wt (*n* = 55) and *IDH*mut (*n* = 15) glioma samples, detected with the HM450K array. Hyper‐ and hypomethylated probes are in pink and light blue, respectively. (B) Genome Browser view at the HOXA cluster to show H3K4me3 enrichment, DNA methylation, the strand‐oriented RNA‐seq signal, and the correlation between these signatures in glioma samples (Glioma) and controls (Brain). (C) On the right panel, heatmap showing CpG site density and their mean methylation level in a ±5 kb window centered on the TSS of HOX genes enriched (upper) or depleted (lower) for H3K4me3 in *IDH*wt glioma samples compared with healthy controls (brain). The ChIP‐seq read density for H3K4me3 is shown on the left panel. (D) Genome Browser view at the *HOXA5* and *HOXD10* loci to show H3K4me3 and DNA methylation enrichment, and the strand‐oriented RNA‐seq signal in glioma samples (Glioma) and controls (Brain). In B to D, publicly available Glioma H3K4me3 data from *IDH*wt‐derived cell lines.

DNA methylation pattern alterations, a hallmark of cancer cells, could contribute to the observed HOX gene deregulation. However, while this mark represses promoter activity, HOX genes are documented to be hypermethylated in glioma samples [18]. Specifically, we previously observed that a class of genes characterized by an aberrant gain of both expression and DNA methylation at their CpG island (CGI) promoter in *IDH*wt glioma samples is precisely highly enriched for HOX genes [22]. High‐throughput DNA methylation analysis of the 55 *IDH*wt samples, using the Infinium HumanMethylation450 (HM450K) BeadChip Arrays, showed DNA hypermethylation at the four HOX clusters, while the surrounding regions tended to be hypomethylated, compared with brain tissue controls (Fig. 4A). Conversely, in *IDH*mut samples, we observed DNA hypermethylation at the HOX gene clusters, although to a lower extent (e.g., 19% along the HOX*A* cluster compared with 25% in *IDH*wt glioma samples), and also in their surrounding regions (Fig. 4A, Table S6). The hypermethylation of the surrounding regions reflected the genomewide trend according to which most of the differentially methylated CpG sites, detected by the HM450K probes, were hypermethylated in *IDH*mut and hypomethylated in *IDH*wt glioma samples (Fig. S2). These findings suggest that HOX cluster targeted hypermethylation is a signature of *IDH*wt glioma.

To understand how DNA hypermethylation and expression gain could coexist, we assessed their correlation in the four HOX clusters. We observed predominantly a positive correlation between DNA hypermethylation and expression, but also short areas of negative correlation both in our samples and in the TCGA cohort. These areas overlapped mostly with unmethylated CGIs among which many contained also a HOX gene transcription start site (TSS) (Fig. 4B, Fig. S3). DNA methylation and H3K4me3 are documented to be antagonist in the genome. To evaluate whether this permissive histone mark could protect TSS from DNA hypermethylation, we assessed its distribution along the four HOX clusters by using publicly available ChIP‐seq data. H3K4me3 specifically marked the areas of negative correlation between DNA methylation and gene expression in *IDH*wt glioma samples, while it was depleted in control samples (Fig. 4B). Accordingly, TSS could be divided into two groups (H3K4me3‐enriched and H3K4me3‐depleted) (Fig. 4C). Analysis of the HM450K data showed that H3K4me3‐enriched TSS was mainly unmethylated, with DNA methylation mostly at their borders (Fig. 4C). Analysis of individual loci in glioma samples using strand‐oriented RNA‐seq data supported the hypothesis that transcription can initiate from these H3K4me3‐marked TSS, embedded in methylated areas (see, for instance, *HOXA5* and *HOXA10*) (Fig. 4D; Fig. S4). However, H3K4me3 enrichment at TSS was not always associated with transcriptional activity, as illustrated for example by the unexpressed *HOTTIP* or the poorly expressed *HOXC5* and *HOXC8* genes (Fig. S4).

At H3K4me3‐depleted TSS, DNA methylation was spread along the entire CGI/promoter, possibly contributing to *HOXB1* lack of expression in glioma, for instance (Fig. S4). However, unlike *HOXB1*, most of the HOX genes in the H3K4me3‐depleted group were highly transcribed, suggesting that transcription from these genes could arise from an alternative TSS. Indeed, analysis of RNA‐seq data showed a transcription signal from H3K4me3‐enriched regions located away from the documented TSS (Fig. 4B, Fig. S4). For instance, *HOXD10* transcription initiated in the *HOXD11* body, leading to a composite transcript (Fig. 4D). Similarly, and as previously proposed [21], a read‐through transcript of *HOXA10* and *HOXA9* might account for the detected *HOXA9* transcription signal because the canonical CGI/promoter of this gene was DNA‐methylated and H3K4me3‐depleted in *IDH*wt glioma samples (Fig. S4).

Altogether, these data suggest that the widespread HOX gene expression in *IDH*wt glioma samples relies on canonical and alternative TSS that escape the DNA hypermethylation observed in all four HOX clusters in these samples.

### 3.5 H3K27me3 loss characterizes HOX clusters in *IDH*wt samples

**Fig. 5:**
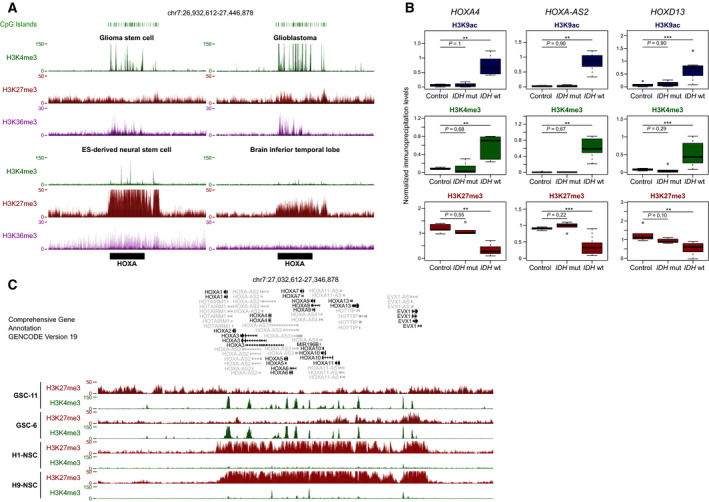
HOX clusters are depleted for H3K27me3 in *IDH*wt glioma and GSC lines. (A) Data mining‐derived ChIP‐seq signal at the HOXA cluster for H3K4me3 (green), H3K27me3 (red), and H3K36me3 (purple) in GSC and *IDH*wt glioma‐derived cell lines (upper panels) and healthy neural stem cell (NSC) and brain tissues (lower panels). (B) ChIP analysis of H3K9ac, H3K4me3, and H3K27me3 at selected HOX genes in control brain samples (*n* = 5), and in *IDH*mut (*n* = 5) and *IDH*wt (*n* = 7) glioma samples. The precipitation level was normalized to that at the *TBP* promoter (for H3K4me3 and H3K9ac) and at the *SP6* promoter (for H3K27me3); **P < 0.01, ***P < 0.001 (Mann–Whitney *U*‐test). (C) ChIP‐seq signal at the HOXA cluster for H3K4me3 (green) and H3K27me3 (red) in two independent GSC lines and in two independent NSC lines.

Besides DNA methylation, H3K27me3 and H3K4me3 histone mark alterations also could be involved in HOX gene deregulation in glioma [16, 19, 21, 22]. Analysis of publicly available ChIP‐seq data showed that their enrichment profiles were significantly altered at the four HOX clusters in *IDH*wt glioma and in GSC samples. The repressive H3K27me3 mark was strongly and specifically enriched at the four HOX clusters in control brain tissue samples and neural stem cells. Conversely, in GSCs and GBM samples, H3K4me3 and the transcription‐associated H3K36me3 marks were enriched at the HOX clusters, underlying their widespread transcriptional reactivation (Fig. 5A, Fig. S5A).

ChIP analysis of randomly selected genes (*HOXA4*, *HOXA‐AS2,* and *HOXD13*) confirmed the marked H3K27me3 loss associated with H3K4me3 and H3K9ac enrichment in seven *IDH*wt glioma samples from our cohort (Fig. 5B, Fig. S5B). Conversely, the five *IDH*mut samples tested were characterized by low H3K4me3 and H3K9ac levels and strong H3K27me3 enrichment, like in controls. Finally, we performed ChIP‐seq analysis of two GSC lines. Compared with neural stem cells (publicly available ChIP‐seq data), in these two lines we observed H3K27me3 loss and H3K4me3 enrichment at the four HOX clusters (Fig. 5C, Fig. S6).

### 3.6 H3K27me3 status recapitulates HOX cluster transcriptional activity

**Fig. 6:**
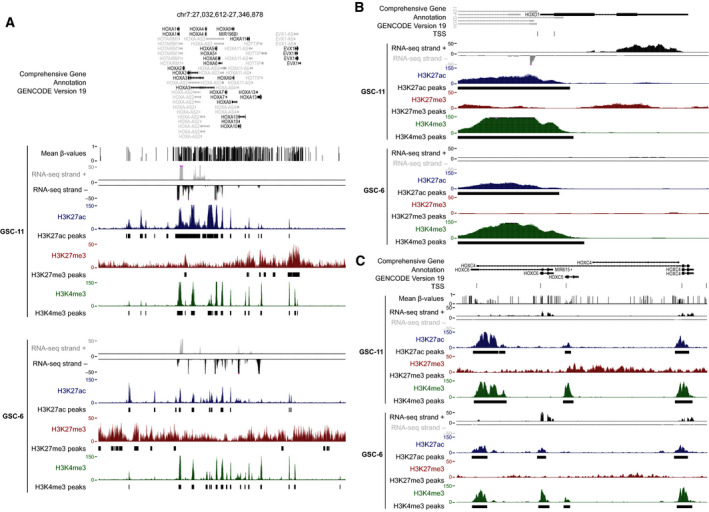
H3K27me3 status recapitulates the transcriptional activity at the HOXA cluster in GSC lines. (A) Genome Browser view at the HOXA cluster to show the strand‐oriented RNA‐seq signal, H3K4me3, H3K27me3, and H3K27ac enrichment, and the DNA methylation signal (only for the GSC‐11 line) in two independent GSC lines. (B) Genome Browser view at the HOXD1 locus to show an integrative view of H3K4me3, H3K27ac, H3K27me3 enrichment, and the strand‐oriented RNA‐seq signal in the two GSC lines. (C) Genome Browser view at the *HOXC4‐C6* locus to show H3K4me3, H3K27ac, H3K27me3 enrichment, DNA methylation (only in the GSC‐11 line), and the strand‐oriented RNA‐seq signal in the two GSC lines.

To further evaluate the relative contribution of histone modifications and DNA methylation changes to HOX gene reactivation in glioma, we analyzed them simultaneously in two IDHwt GSC lines, CSG‐6 and CSG‐11. The extent of transcriptional reactivation at the four HOX clusters differed between cell lines. For example, HOXA cluster gene reactivation was widespread in GSC‐6 cells, but was restricted to the telomeric part of the cluster in GSC‐11 cells (Fig. 6A, Fig. S6A). Like in glioma samples (Fig. 4), we observed a DNA methylation gain (analyzed only in the GSC‐11 line) at all four clusters, irrespective of the gene transcriptional status. Similarly, H3K4me3 (permissive mark) was enriched (compared with control neural stem cells) at CGIs in all clusters, irrespective of the transcriptional activity of the associated genes. This analysis highlighted that the H3K27me3 status recapitulated the transcriptional activity. For instance at the HOXA cluster, the widespread gene expression observed in GSC‐6 cells correlated with H3K27me3 loss in the entire cluster. Conversely, in GSC‐11 cells, H3K27me3 was enriched only in the centromeric part of the HOXA cluster, where the concomitant enrichment for H3K4me3 led to a bivalent signature at most CGI/promoters. Accordingly, HOXA genes located in this area were repressed. On the other hand, the telomeric part of the HOXA cluster was characterized by H3K27me3 depletion, and CGI/promoters were enriched only in H3K4me3, a signature that overlapped with transcriptional activity (Fig. 6A). As previously observed [32], loss of H3K27me3 was coupled with gain of H3K27ac that can further facilitate transcriptional activation. We obtained similar results also for the other three clusters (Fig. S6A). These findings suggest that absence of bivalency, due to H3K27me3 loss, at HOX CGI/promoters could be one of the main mechanisms to explain aberrant HOX gene expression in GSC cells. However, at some rare genes, absence of bivalency was not sufficient to promote aberrant expression. For instance, the CGI/promoter of the *HOXD1* gene showed a H3K4me3‐only signature in both cell lines, but this gene was expressed only in GSC‐11 cells (Fig. 6B). This observation could be explained by cell line‐specific post‐transcriptional regulations. Moreover, bivalency was not the only signature associated with repressed genes. For instance, the CGI/promoters of *HOXB1* and *HOXC12*, two genes that were not expressed in glioma samples, were marked by a combination of H3K27me3‐only and DNA methylation (Fig. S6B, Fig. 1C). This integrative approach also confirmed that the use of alternative TSS contributed to HOX gene derepression. For example, *HOXC6* was expressed in both cell lines. However, in GSC‐6 cells, the short isoform was mainly expressed from its canonical promoter that is associated with an active H3K4me3/H3K27ac signature only in this cell line. In GSC‐11 cells, this canonical promoter is DNA methylated. Combined analysis of RNA‐seq and ChIP‐seq data suggested that in GSC‐11 cells*, HOXC6* transcription rather initiated from the H3K4me3/H3K27ac‐enriched alternative promoter overlapping with that of *HOXC4*, leading to the production of the longer form (Fig. 6C). Similar observations were also made at *HOXA2* and *HOXB8* loci, for instance (Fig. S7).

## 4 Discussion

Here, we investigated the extent and molecular bases of HOX gene deregulation in adult diffuse glioma.

First, our data confirmed previous observations made in tumors classified according to the 2007 WHO criteria (i.e., GBM and lower grade glioma) that many HOX genes are deregulated in glioma and specifically in GBM [14, 15, 16, 18, 21]. By analyzing glioma samples classified according to the most recent WHO classification [2], the present study extended these observations and provided, for the first time, a comprehensive view of gene deregulation in the different clusters. Our data showed that widespread HOX transcriptional alteration, affecting both sense and antisense transcripts, is a characteristic of *IDH*wt glioma samples. Among the 57 HOX transcripts (39 sense and 18 antisense), on average 32.5 (median *n* = 37) were deregulated in each of the 177 *IDH*wt samples analyzed (our cohort + the TCGA cohort) and 11.75 (median *n* = 11) in each of the 423 *IDH*mut samples. In line with the absence of *HOX* gene expression in healthy brain, most HOX genes were upregulated, and only *HOXD1* and *HOXD‐AS1*, which are expressed in healthy brain, were downregulated in *IDH*wt samples. This observation suggests that deregulation affects all four HOX clusters and does not follow the coordinated collinear expression observed during normal embryonic development [18]. Given the key role of HOX transcription factors during normal development, it is reasonable to hypothesize that HOX gene upregulation contributes to gliomagenesis. Accordingly, all members, but *HOXD10*, of the core HOX signature of *IDH*wt samples (i.e., upregulation of *HOXA1, A5, A6, A7, A10, D9,* and *D10*) have an oncogenic role in gliomas, by promoting cell viability and migration or by reducing cell death of GBM cell lines [18, 19, 33, 34]. Besides the widespread HOX gene upregulation, our study also identified four transcripts (*HOXB1*, *HOXC12*, *HOTTIP,* and *HOXB‐AS4*) that were not reactivated. *HOXC12* has never been found to be aberrantly expressed in solid tumors [17, 35], suggesting that *HOXC12*‐overexpressing cancer cells might be counterselected.

Our study questions the relationship between aberrant transcription and DNA methylation in cancer cells. In a previous genomewide study, we observed a gain of DNA methylation at the CGI/promoters of about 16% of ectopically expressed genes (mainly HOX genes and homeobox genes) in *IDH*wt samples [22]. Here, we confirmed and refined this observation by showing that *IDH*wt samples present a marked gain of DNA methylation along all HOX gene clusters, irrespective of their transcriptional status. By focusing on the *HOXA10* gene, Kurscheid *et al*. [21] proposed that in aggressive glioma, DNA methylation is a protective mechanism to counteract the effect of chromosome 7 gain at the HOXA cluster. Specifically, hypermethylation of the HOXA cluster could compensate CNV at this cluster in some tumors, leading to low expression level of HOX genes. Conversely, key CpG sites located in the HOXA locus could escape this hypermethylation in other tumors, leading to high expression of HOX genes [21]. Our observations suggest a more complex mechanism at the four HOX clusters, irrespective of CNV, and through the use of alternative promoters. In line with the findings on *HOXA10* [21], we observed that at many HOX genes, ectopic expression was associated with CGI/promoters that gained methylation at their borders, whereas the H3K4me3‐marked TSS was methylation‐free, thus allowing transcription. Other CGI/promoters were fully methylated. For instance, we found this signature at the *HOXB1* and *HOXC12* genes, providing a mechanism to explain their silencing in all analyzed samples. However, unlike these two rare examples, extensive methylation of the CGI/promoter of other HOX genes was often associated with the use of an alternative promoter, as previously reported for various genes in prostate cancer cell lines [36] and the *DCLK1* gene in human colon adenocarcinoma [37]. This suggests the frequent use in cancer of an alternative promoter when the main CGI/promoter is hypermethylated. Interestingly, this mechanism might contribute to the expression pattern and particularly to splice variant variability among samples. Indeed, we observed that a given gene could be transcribed from its main CGI/promoter in some samples, and from an alternative promoter in other samples where the main promoter was fully methylated, further supporting a causal link between canonical promoter methylation and use of an alternative promoter.

Our main observation, obtained through a refined integrative analysis of GSC lines, is that unlike DNA methylation and H3K4me3 level, the repressive H3K27me3 mark status (i.e., presence/absence) best recapitulated the transcriptional activity along the four HOX clusters. Our data suggest that a bivalent signature (H3K4me3 and H3K27me3) at CGI/promoters maintains repressed a subset of HOX genes in glioma cells, while H3K4me3 alone marks the promoter of active HOX genes. Accordingly, alterations in the pathways controlling these two marks have been involved in HOX gene transcriptional deregulation in glioma. For instance, the H3K4me3 methyltransferase mixed lineage leukemia (MLL) is required for *HOXA10* activation in GSCs [19], while the PI3K pathway, by mediating suppression of H3K27me3, contributes to HOXA cluster derepression in GBM cell lines [16]. Although aberrant hypermethylation of HOX genes is considered as a pan‐cancer signature [38], our data suggest that aberrant expression of HOX genes in various cancer types [17] could mainly rely on alterations in H3K27me3 regulation. Noteworthy, H3K4me3 marks by default most HOX CGI/promoters in GSC lines, and this mark is maintained also in glioma samples, but not in healthy brain samples. This signature is closer to that observed in human stem cells and to a lesser extent in neural precursor cells, where a bivalent signature marks most HOX GCI/promoters. This observation argues for a stem cell origin of GSCs rather than from the de‐differentiation of adult brain cells.

Our study suggests that H3K27me3 loss in the four HOX clusters, and specifically at bivalent CGI/promoters of ‘masters’ HOX genes, is the main cause of widespread *HOX* gene upregulation in glioma cells. Besides alterations in the machinery and pathways controlling H3K27me3 [16], cancer‐associated genomewide hypomethylation might be instrumental in its loss. Studies in the mouse revealed that induced widespread DNA methylation depletion triggers H3K27me3 redistribution [39, 40] that in turn leads to H3K27me3 loss and ectopic expression of a subset of polycomb target genes, including HOX genes [40]. Altogether, our observations support a model whereby genomewide hypomethylation leads to drastic H3K27me3 loss (and localized DNA methylation gain) at HOX clusters, facilitating the reactivation of a group of HOX genes that are bona fide polycomb target genes. Widespread HOX gene reactivation would be reinforced by master ‘HOX’ transcription factors that can target other HOX genes. Our study identified *HOXA11*, *HOXC11, HOXC13, HOXD11,* and *HOXD12* as putative upstream factors that might activate other HOX genes, mainly in the same cluster. Additional studies are now required to test this model and evaluate whether these five products are true ‘master’ factors or if they are downstream of an unknown key regulatory factor. Nevertheless, these HOX factors are relevant candidates to support GSC tumorigenic potential.

## 5 Conclusions

Our study provides the first comprehensive description of the epigenetic changes at HOX clusters in glioma and their relative contribution to their transcriptional changes. It showed how DNA hypermethylation and gene overexpression can coexist and suggests that loss of H3K27me3 along the HOX clusters is the main driving force of their widespread transcriptional alteration in *IDH*wt samples. It highlighted the complexity of HOX gene expression pattern in patients where the usage of alternative promoters contributes to splice variant variability among samples.

## Conflict of interest

The authors declare no conflict of interest.

## Author contributions

PA initiated and supervised the study. ELB, FC, AF, and PA designed the study. P‐OG and LKT isolated and characterized the GSC lines. EC and PV characterized the glioma samples. ELB, FC, CV‐B, IV, and AF performed the experiments. FC performed the bioinformatic analyses. ELB, FC, CV‐B, IV, BMC, AF, and PA analyzed the data. ELB produced the figures with FC's and PA's input. PA wrote the paper. All authors read and approved the final manuscript.

### Peer Review

The peer review history for this article is available at https://publons.com/publon/10.1002/1878‐0261.12944.

## Supporting information


**Fig. S1.** Widespread reactivation of HOX *genes* in *IDH*wt glioma samples.
**Fig. S2.** Genome‐wide DNA methylation pattern.
**Fig. S3.** Integrative view of the molecular signatures at the HOXB, C and D clusters.
**Fig. S4.** Representative examples of the correlation between DNA methylation and expression at HOX genes in *IDH*wt glioma samples.
**Fig. S5.** HOX clusters are depleted for H3K27me3 in *IDH*wt glioma and GSC lines.
**Fig. S6.** H3K27me3 status recapitulates the transcriptional activity at the HOX clusters in GSCs.
**Fig. S7.** Use of an alternative TSS contributes to HOX gene derepression.

**Table S1.** Demographic and clinical features of patients with glioma.

**Table S2.** Primer list.

**Table S3.** HOX gene expression levels in *IDH*wt glioma samples compared with controls.

**Table S4.** Percentage of samples that express the indicated HOX transcripts (log10(fold‐change) compared with the housekeeping genes *PPIA*, *TBP* and *HPRT*1: ≥ ‐2 in our cohort; log10(FPKM) ≥ ‐1 in the TCGA cohort).

**Table S5.** Correlation between CNV and gene expression at the HOXA cluster.

**Table S6.** Comparison of the methylation values at the HOX clusters among control brain samples, and *IDH*wt and *IDH*mut glioma samples.

## Data Availability

Data on the two GSC lines generated for this study were submitted to NCBI Gene Expression Omnibus (GEO; https://www.ncbi.nlm.nih.gov/geo/) under the following accession numbers: GSE161175 for the Epic DNA methylation data, GSE161438 (Ribo‐zero) and GSE161437 (mRNA) for the oriented RNA‐seq data, and GSE161436 for Chip‐seq data. CNV data generated using glioma samples for this study were submitted under the GSE161275 accession number.
