# Supplementary material for: Widespread overexpression from the four DNA hypermethylated HOX clusters in aggressive (IDHwt) glioma is associated with H3K27me3 depletion and alternative promoter usage
Source: Mol Oncol. 2021 May 2;15(8):1995–2010. doi: 10.1002/1878-0261.12944 (PMC8334257; doi:10.1002/1878-0261.12944)
Supplement: Supplementary file 1 — Fig. S1. Widespread reactivation of HOX genes in IDHwt glioma samples. Fig. S2. Genome‐wide DNA methylation pattern. Fig. S3. Integrative view of the molecular signatures at the HOXB, C and D clusters. Fig. S4. Representative examples of the correlation between DNA methylation and expression at HOX genes in IDHwt glioma samples. Fig. S5. HOX clusters are depleted for H3K27me3 in IDHwt glioma and GSC lines. Fig. S6. H3K27me3 status recapitulates the transcriptional activity at the HOX clusters in GSCs. Fig. S7. Use of an alternative TSS contributes to HOX gene derepression. [file MOL2-15-1995-s001.pdf]

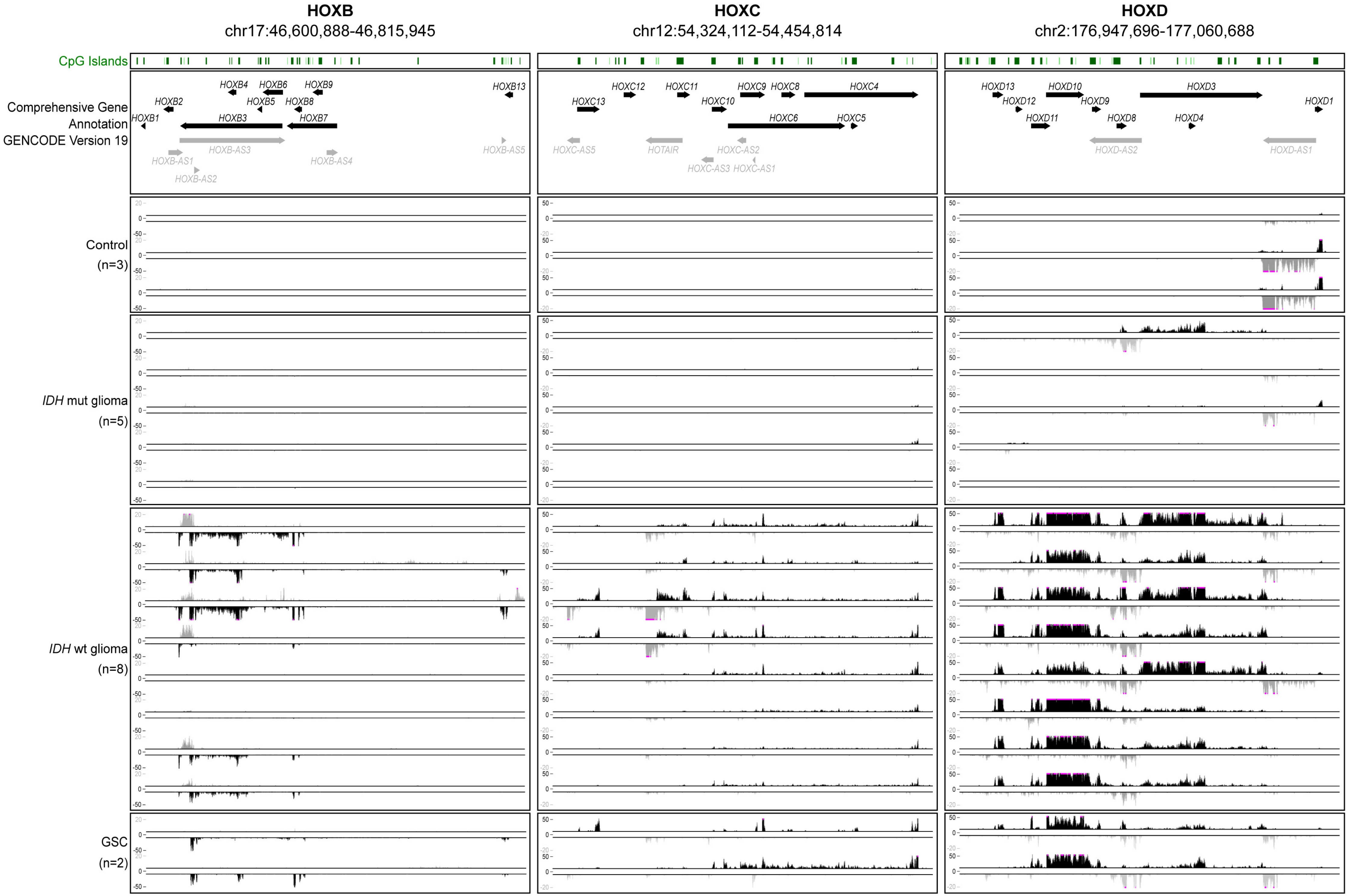

Supp Figure1: Widespread reactivation of HOX genes in IDHwt glioma samples

Strand-oriented RNA-seq signal along the HOXB (A), C (B) and D (C) clusters in brain controls, IDHmut and IDHwt glioma and GSC samples.

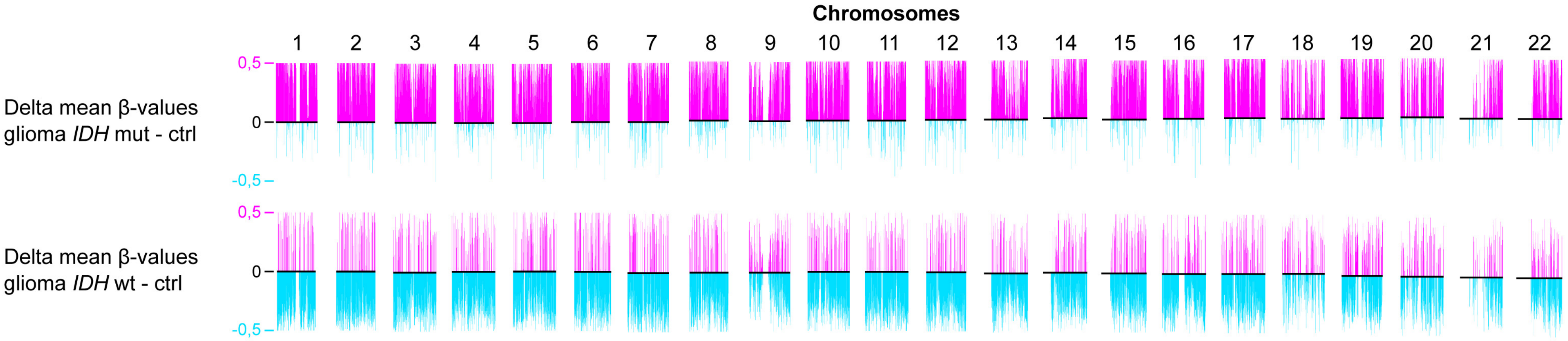

Supp figure 2: Genome-wide DNA methylation pattern

DNA methylation changes (compared with healthy brain controls) in each chromosome in IDHwt (n=55) and IDHmut (n=15) glioma samples, detected with the HM450K array. Hyper- and hypo-methylated probes are in pink and light blue, respectively. A summary of the proportions of hypo- and hypermethylated probes is given in Table S6.

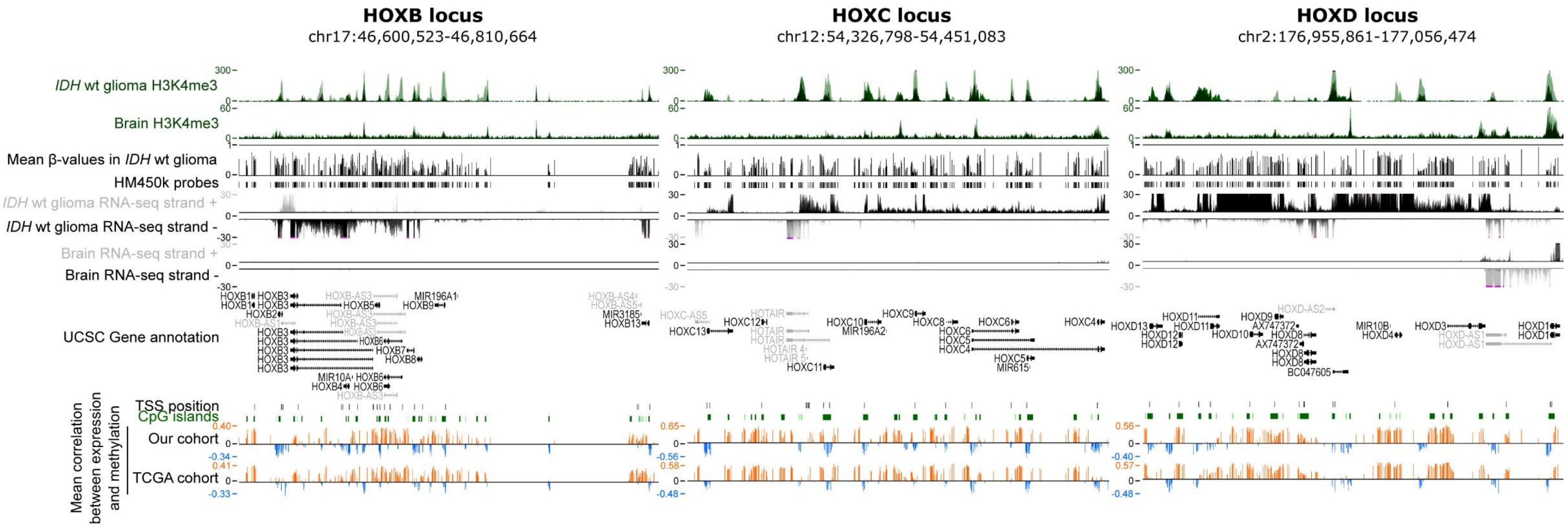

Supp figure 3: Integrative view of the molecular signatures at the HOXB, C and D clusters

Genome Browser view at the HOXB, HOXC and HOXD clusters to show H3K4me3 enrichment, differential DNA methylation, the strand-oriented RNA-seq signal, and the correlation of these signatures**.** The publicly available Glioma H3K4me3 signals are from *IDH*wt-derived cell lines.

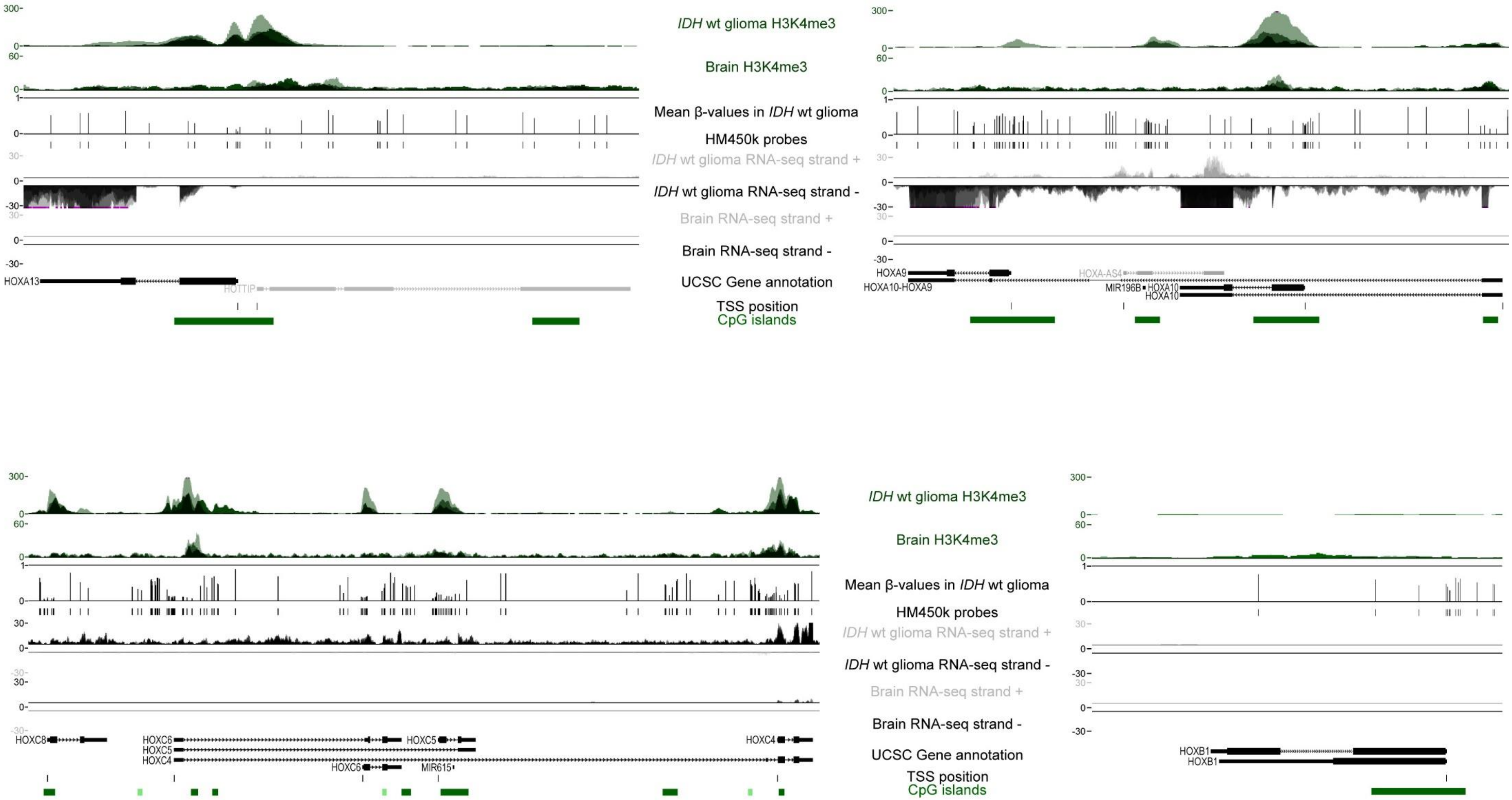

Supp figure 4: Representative examples of the correlation between DNA methylation and expression at HOX genes in *IDH*wt glioma samples.

Genome Browser view of the *HOXA10*, *HOTTIP*, *HOXC5* and *HOXB1* loci to show H3K4me3 enrichment, DNA methylation, and the oriented RNA-seq signal. The H3K4me3 signals are from publicly available data of *IDH*wt-derived cell lines. For *HOXA10*, transcription initiates from a H3K4me3-marked TSS in an embedded methylated area. *HOTTIP* and *HOXC5-HOXC8* are not transcribed although their TSS are DNA unmethylated and H3K4me3-enriched. *HOXB1* is a (rare) example of not expressed gene with a DNA methylated and H3K4me3-poor TSS. *HOXA9* transcription occurs through an alternative promoter.

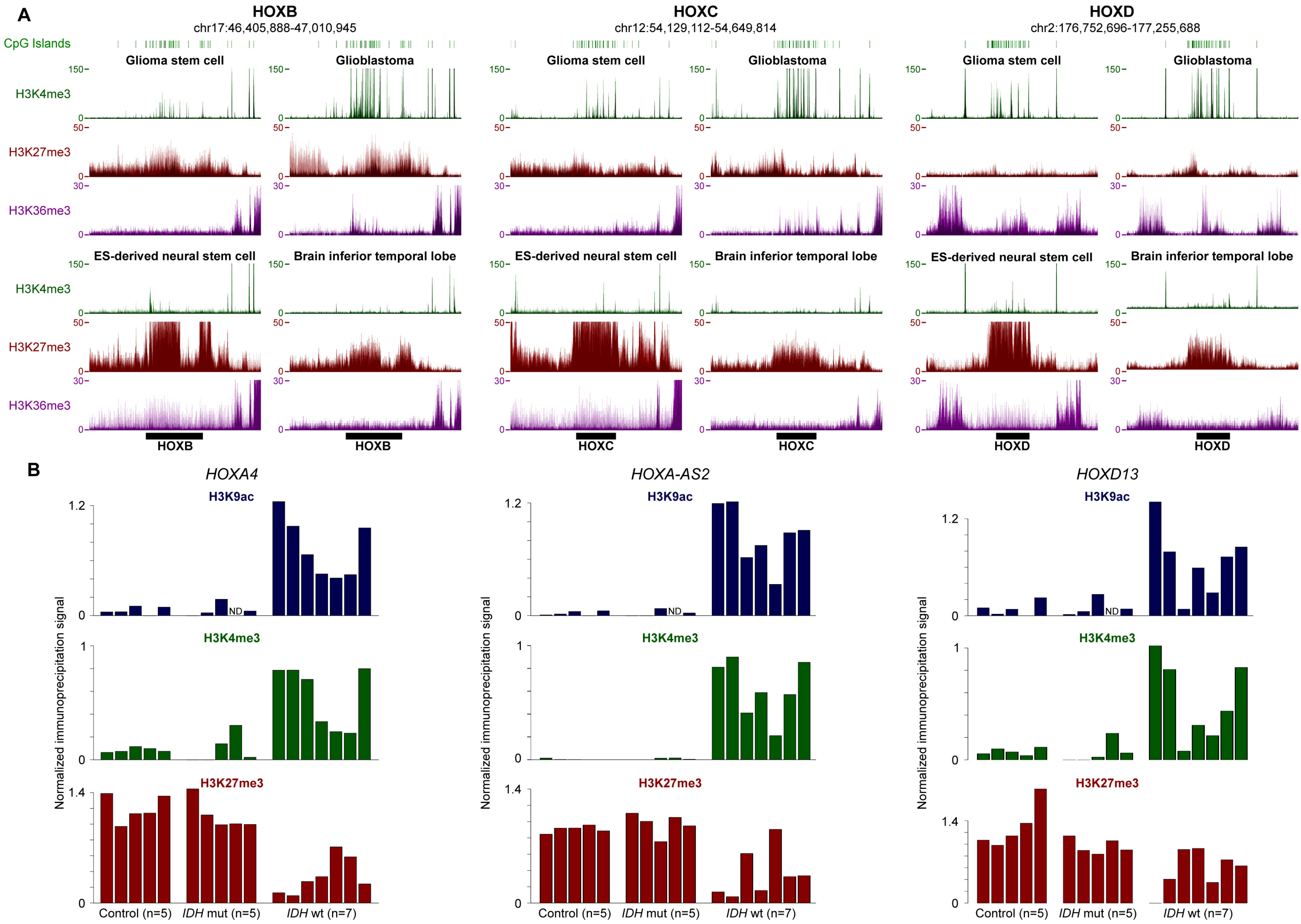

Supp Figure 5: HOX clusters are depleted for H3K27me3 in IDHwt glioma and GSC lines

A) Data mining–derived ChIP-seq signal at the HOXB, HOXC and HOXD clusters for H3K4me3 (green), H3K27me3 (red) and H3K36me3 (purple) in GSC and IDHwt glioma-derived cell lines (upper panels) and healthy neural stem cells and brain tissues (lower panels). B) ChIP analysis of H3K9ac, H3K4me3, and H3K27me3 at selected HOX genes in control brain samples (n=5), IDHmut (n=5) and IDHwt (n=7) glioma samples. The values obtained for each sample are shown. The precipitation level was normalized to that at the TBP promoter (for H3K4me3 and H3K9ac) and at the SP6 promoter (for H3K27me3).

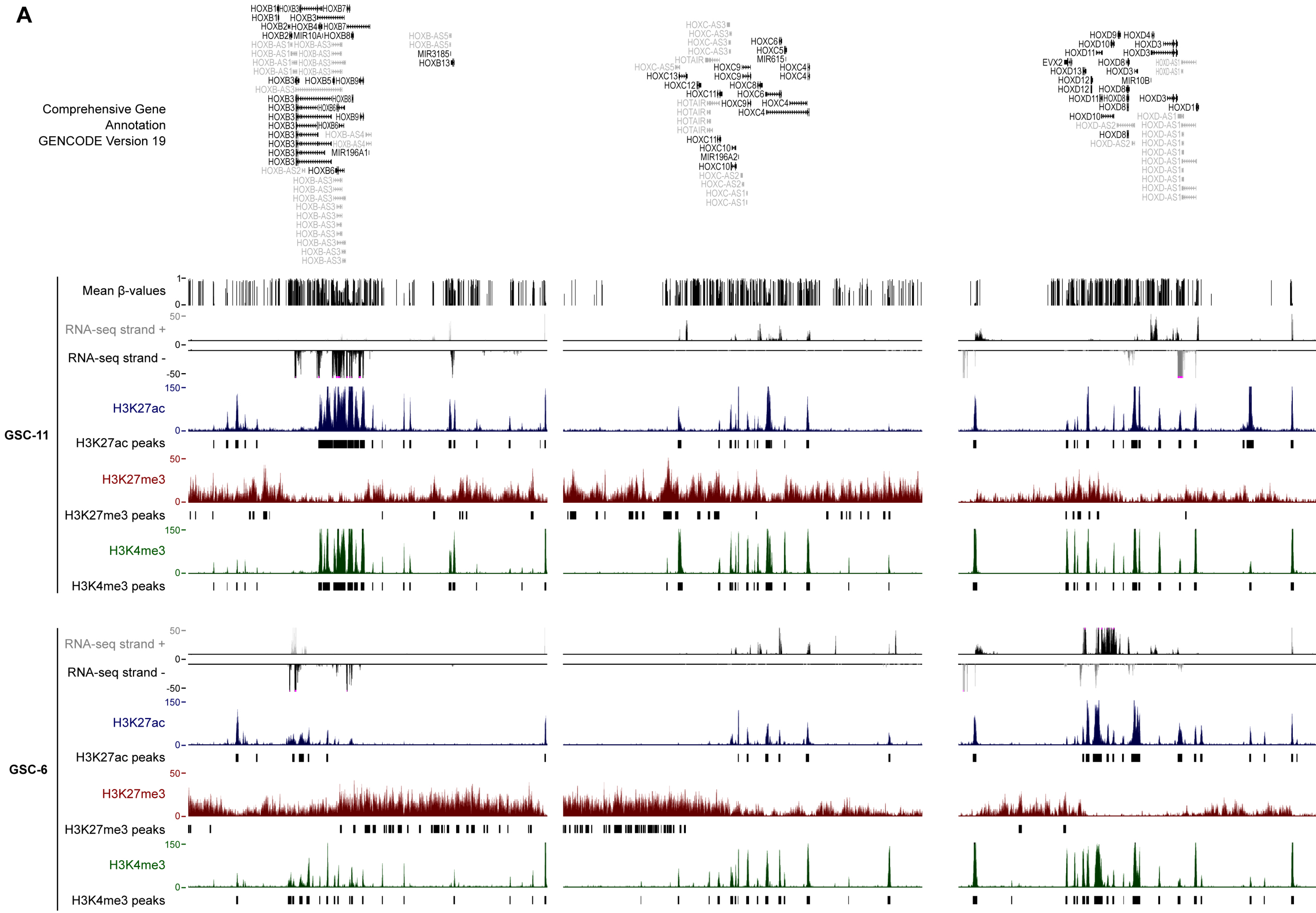

Supplementary Figure 6: H3K27me3 status recapitulates the transcriptional activity at the HOX clusters in GSCs

A) Genome Browser view at the HOXB, HOXC and HOXD clusters to show the strand-oriented RNA-seq signal, H3K4me3, H3K27me3 and H3K27ac enrichment, and the DNA methylation signal (only for the GSC-11 line) in two independent GSC lines.

B

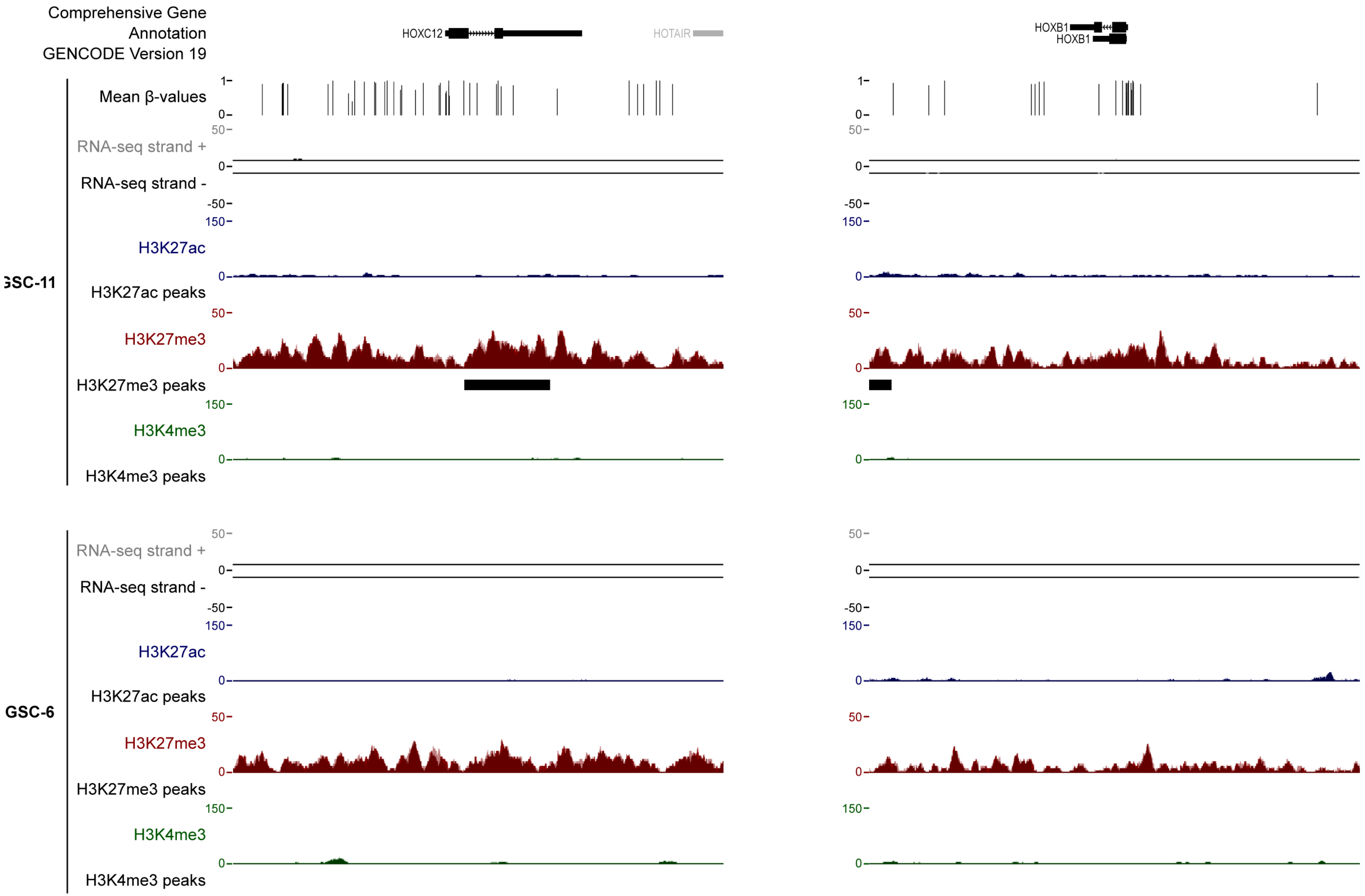

Supplementary Figure 6: (Continued)
B) Details for HOXC12 and HOXB1 loci.

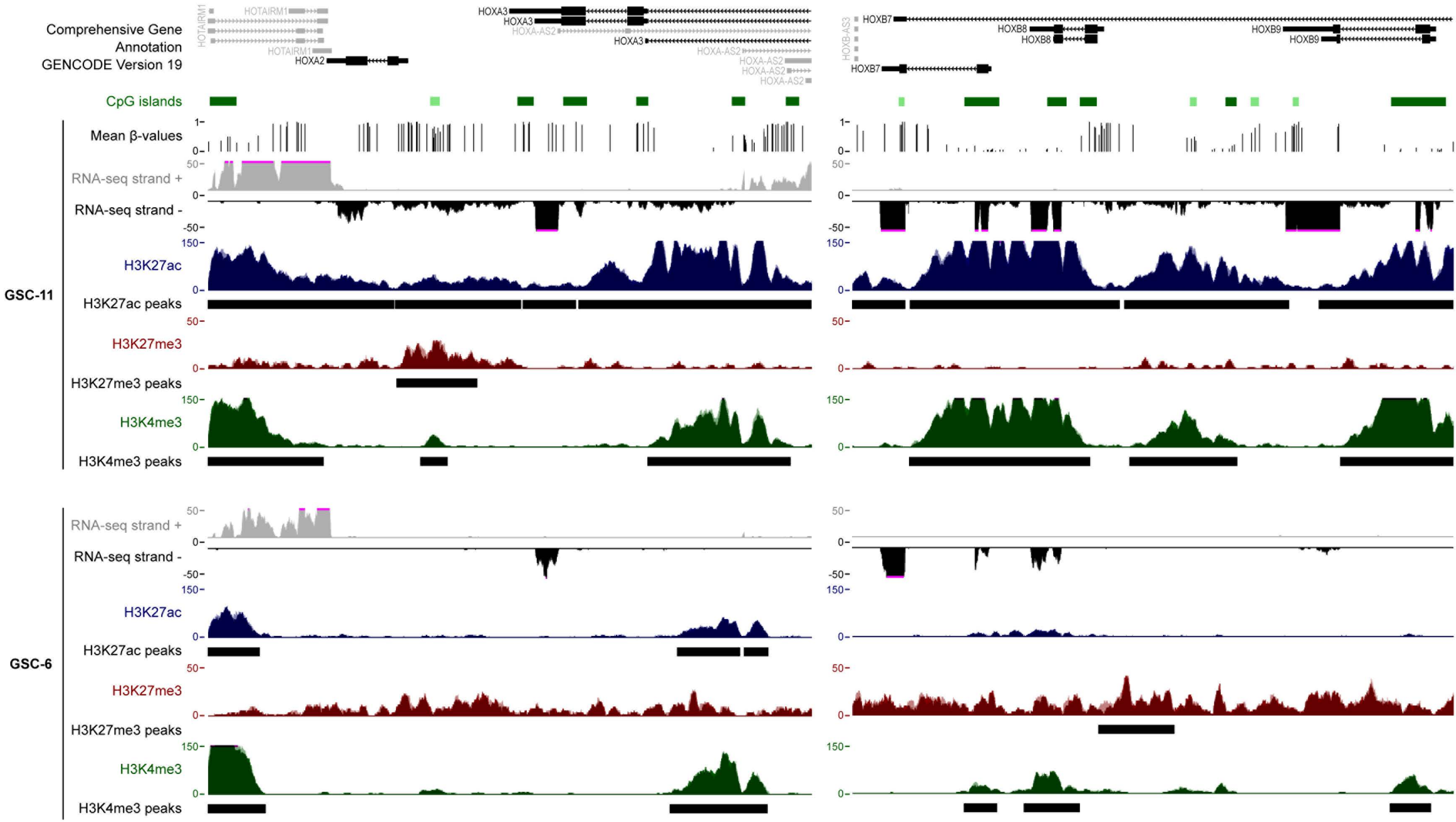

Supplementary Figure 7: Use of an alternative TSS contributes to HOX gene derepression

Genome Browser view of the HOXA2 and HOXB8 loci to show, CpG islands, the strand-oriented RNA-seq signal, H3K4me3, H3K27me3 and H3K27ac enrichment, and the DNA methylation signal in the GSC-11 cell line. These two loci illustrate alternative promoter usage when the canonical CGI/promoter is DNA-methylated.
