# Supplementary material for: Widespread overexpression from the four DNA hypermethylated HOX clusters in aggressive (IDHwt) glioma is associated with H3K27me3 depletion and alternative promoter usage
Source: Mol Oncol. 2021 May 2;15(8):1995–2010. doi: 10.1002/1878-0261.12944 (PMC8334257; doi:10.1002/1878-0261.12944)
Supplement: Supplementary file 2 — Table S1. Demographic and clinical features of patients with glioma. [file MOL2-15-1995-s004.docx]

| Characteristics | Mutated IDH1 patients (n=15) | | WT IDH1 patients (n=55) | |
| --- | --- | --- | --- | --- |
|  | Nb of patients | % | Nb of patients | % |
| Sex |  |  |  |  |
| Female | 5/15 | 33% | 23/55 | 42% |
| Male | 10/15 | 67% | 32/55 | 58% |
| Age at diagnosis (years) |  |  |  |  |
| Mean (SD) | 40 (10.4) | | 60 (10.2) | |
| Range | [26-63] | | [29-80] | |
| 7p+/10q- (CNV) | 0/13 | 0% | 23/34 | 68% |
| unknowm status 7p10q | 2/15 | 13% | 21/55 | 38% |
| Tot 1p19q codeleted (FISH+INA+CNV) | 9/14 | 64% | 2/55 | 4% |
| unknowm status 1p19q | 1/15 | 7% | 0/55 | 0% |
| *MGMT* promoter methylation (>8%) | 14/15 | 93% | 25/55 | 45% |

| Resection |  |  |  |  |
| --- | --- | --- | --- | --- |
| Total | 6/15 | 40% | 23/55 | 42% |
| Partial | 7/15 | 47% | 31/55 | 56% |
| Large biopsy | 2/15 | 13% | 1/55 | 2% |

| Post-surgery treatment |  |  |  |  |
| --- | --- | --- | --- | --- |
| R + TMZ = "Stupp" | 5/15 | 34% | 41/55 | 75% |
| Radiotherapy only | 2/15 | 13% | 3/55 | 5% |
| Chemotherapy only | 4/15 | 27% | 2/55 | 4% |
| No treatment | 2/15 | 13% | 4/55 | 7% |
| unknown | 2/15 | 13% | 5/55 | 9% |
| Overall survival (years) |  |  |  |  |
| Median | Not reached | | 1.25 | |
| Range | [0.1-6.6] | | [0.1-3.8] | |

- **Supplementary Table S1** (Doc): Demographic and clinical features of patients with glioma

Supplemental table S1: Demographic and clinical features of the 70 patients with glioma. The 7p+/10q- status (gain in chromosome 7p and loss in chromosome 10q) was assessed by copy number variation (CNV) analysis. The 1p19q co-deletion status was determined by merging data obtained by fluorescence in situ hybridization (FISH), alpha-internexin immunostaining (INA) and CNV analysis. *MGMT* promoter methylation is the mean of the methylation of five CpG sites located between the hg19 coordinates 131265507 and 131265526. The “Stupp” post-surgery treatment combines radiotherapy (R) and temozolomide (TMZ)-based chemotherapy. SD: standard deviation
